# Smart Glove for Maintenance of Industrial Equipment

**DOI:** 10.3390/s25030722

**Published:** 2025-01-25

**Authors:** Natalia Koteleva, Aleksander Simakov, Nikolay Korolev

**Affiliations:** 1 Department of Automation of Technological Processes and Production, Empress Catherine II Saint Petersburg Mining University, 2, 21 Line of Vasilyevsky Island, 199106 St. Petersburg, Russia; koteleva_ni@pers.spmi.ru (N.K.); simakov_as@pers.spmi.ru (A.S.); 2 Educational Research Center for Digital Technologies, Empress Catherine II Saint Petersburg Mining University, 2, 21 Line of Vasilyevsky Island, 199106 St. Petersburg, Russia

**Keywords:** wearable devices, smart glove, maintenance and repair, IoT devices, maintenance software services

## Abstract

Maintenance and service are important tasks for any industrial enterprise. This article presents a methodology for technical maintenance that employs a smart glove equipped with tactile sensors, an electronic unit responsible for processing and transmitting information, and a unit designed to interpret the results. Tactile sensors are graphene-based. The main idea of the method is to use sensors to record the strength of contact between the operator’s fingertips and the equipment. Afterwards, these values are recorded, transferred to processing, and the output signal from the sensors is compared with the steps of various repair works. The work contains methods for creating each component of the glove, their effectiveness is evaluated, and experiments are described to assess the feasibility of using the developed device for the maintenance and repair of equipment. The device discussed in this work is a wearable device. The obtained results demonstrate the applicability of the smart glove for equipment maintenance and repair.

## 1. Introduction

Industrial equipment and its technical condition play essential key roles in the operation of any industrial enterprise [1,2]. In the mining, metallurgical, oil and gas, and electric power industries, particular emphasis is placed on the management of equipment maintenance processes [3,4]. Digital technologies play a significant role in this context, with various sensors [5,6], machine vision [7,8], special communication channels [9,10], predictive analytics systems [11,12], and equipment technical resource assessment [13,14]. A crucial aspect in the utilisation of these technologies is the evaluation of their effectiveness and the suitability of their application in specific scenarios [15,16]. Many tasks are accomplished in maintenance and repair management. The following aspects are of the greatest importance: the identification of equipment maintenance and repair actions in real time [17,18], the comparison of personnel actions with the so-called equipment repair map [19], the evaluation of the length of each action [20,21], and the registration of downtime in maintenance and repair areas [22,23], etc. At present, just a few of these tasks have been automated. Meanwhile, studies such as [24,25] show that factors related to work and its organization significantly influence the causes of human errors, as do environmental, individual, and technical factors. A number of researchers are committed to the automation of organisational support and the establishment of flexible policies for the maintenance and repair of equipment, with the capacity to adapt to changing conditions [26,27]. As an example, in the work in [28], an RL (reinforcement learning)-based agent is developed to autonomously determine the optimal time to perform corrective or preventive maintenance. The other research utilises video and photographic recording of the repair process. This study [29] presents an approach for detecting and assessing the wear of wind turbine components (carbon brushes) based on brush image analysis. A huge volume of work covers the use of wearable devices in maintenance. Wearable devices include different sensors, augmented reality glasses, handheld scanners, and others [30,31]. Also, this paper [32] shows both the use of augmented reality technology in performing repairs and a solution to one of the technology’s most pressing issues; namely, the creation of automated maintenance instructions. One study [33] presents the combined use of video and wearable sensors attached to the athlete’s body. The authors made it possible to collect real-time data on the movement of athletes and to provide feedback for the training process. This study is indirectly related to current research; however, the video and sensor combination is of particular interest and has the potential to be used for maintenance management. Another group of studies demonstrates the application of wearable devices in industry, in particular, in providing human location, action recognition, and gesture evaluation. As an example, a recent paper reviews the application of smart insoles for such tasks [34], a smart vest [35], and a smart helmet [36]. The work in [37] presents the development of an augmented reality system for training heavy equipment operators for a coal mine in the USA. Specifically, gloves as wearable devices have been extensively researched, with a multimodal action-oriented warning glove being a well-documented application [38]. Furthermore, this work [39] puts forward the concept of utilising a smart glove in combination with a Phalange-based Triboelectric Sensor (PTS) to facilitate the training of an Embodied Artificial Intelligence (EAI) system. This glove uses the Phalange-based Triboelectric Sensor (PTS). It integrates a linkage mechanism with a double-layer electrode design. A vast number of works are focused on the study of the cognitive–psychological state of personnel when using wearable devices [40,41]. In particular, this study [42] evaluates the impact of using wearable devices on an operator performing manual material handling tasks. The paper illustrates that glasses are considered the most impractical, least comfortable, and most effective devices for manual assembly tasks.

According to the present article, a methodology is proposed for the control of maintenance and repair processes of industrial equipment by means of a smart glove. The device under discussion is a wearable, comprising graphene-based [43] tactile sensors, an electronic unit for information processing and transmission, and a software service for the real-time recording of values, the determination of processes, and the prescription of maintenance and repair actions.

## 2. Materials and Methods

The design of a smart glove is composed of several stages, including the selection of sensors and technologies that are able to capture the state and movements of the hand and fingers. Common solutions in glove design include the installation of piezoelectric sensors that are able to modify their resistance when subjected to stretching and compression. These sensors must be installed on specific parts of the palm that are most sensitive to changes during manual work. However, such solutions are associated with the necessity of precise adjustment of the sensors to the specific palm of a particular person. This requirement for individualization is a significant challenge in manufacturing. Our paper proposes sensors that are capable of detecting manual effort. The placement of these sensors is straightforward, as they can be positioned beneath the fingertip. This condition can be easily fulfilled by placing the glove on the correct side. Therefore, when designing the glove, it is necessary to fix this sensor on one side of the glove. It has to be positioned beneath the finger pad. The subsequent stage is to design and develop the information processing and transmission unit, which will include a microcircuit with a microcontroller for processing signals. The processing frequency should be optimised to ensure that the data collected from the sensor are processed and transmitted over the wireless network, and then the result received. The following requirements are taken into consideration when designing the information processing and transmission unit: the unit should be lightweight and compact in size to easily fix it on the wrist or the outer side of the palm; it should be energy-saving, with a long-lasting and sufficient power supply source; and battery replacement should be simple. The next step involves the architecture and design of the results interpretation unit. This unit has a number of requirements, primarily the capacity to receive signals via a wireless communication channel as a primary medium for the information and transmission processes. Secondly, the computing power needs to be adequate to ensure the effective processing of the obtained results, following interpretation with the aid of machine learning models. Thirdly, the unit should possess the capacity to implement a user interface for interaction and have resources to create a database, fix results, and keep records. Finally, this unit should be able to integrate other information coming from third-party systems (for example, industrial control systems) and also self-integrate into different systems.

**Figure 1:**
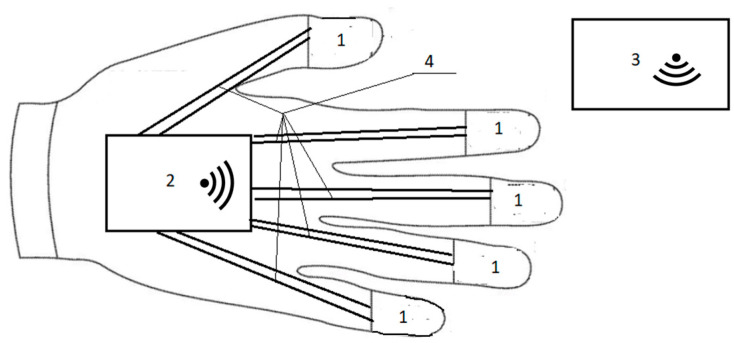
Device overview.

Figure 1 shows a smart glove overview for maintenance and repair.

The glove includes tactile sensors (1), an electronic information processing and transmission unit (2), a results interpretation unit (3), and a pair of Teflon-insulated copper wires (4) for connecting the sensors to the electronic information processing and transmission unit.

**Figure 2:**
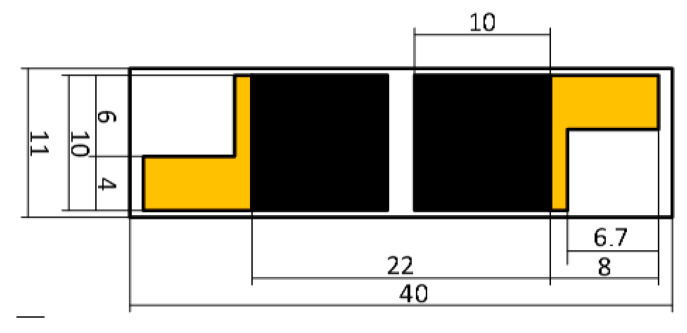
Scheme of the first sensor part (LIG).

The fabrication of the tactile sensor used in the glove was primarily based on the findings in paper [44], with certain modifications. The tactile sensor consists of two parts: laser-induced graphene (LIG) and reduced graphene oxide (rGO) [45]. In the first part of the fabrication process, foil-coated polyimide was used. The application of photolithography to the surface of the foil polyimide facilitates the formation of conductive areas. These areas are intended for two principal functions; firstly, to serve as solder points for connecting wires, and secondly, to facilitate contact with graphene. It is necessary to point out that during the laser induction of graphene it has contact with the foil. Flexible, heat-resistant copper wires with Teflon insulation, with a cross-section of 0.07 mm^2^, were soldered to each foil part. Figure 2 shows the scheme of the first sensor part.

The second part of the sensor, reduced graphene oxide (rGO), is manufactured in the following manner. A woolly cotton fabric with a density of 175 g/m^2^ and a thickness of 1 mm is soaked in a water suspension of graphene oxide for a day (rGO concentration 2mg/mL, multilayer rGO content not less than 80%, elemental composition of graphene oxide C (%)—46.0–47.0, O (%)—49.0–50.0, H (%)—2.5–3.0, N (%)—0.0, S (%)—<1.0) and afterwards calcined in a furnace at 200 °C. The resulting fabric is cut into squares with a size of 10 mm.

**Figure 3:**
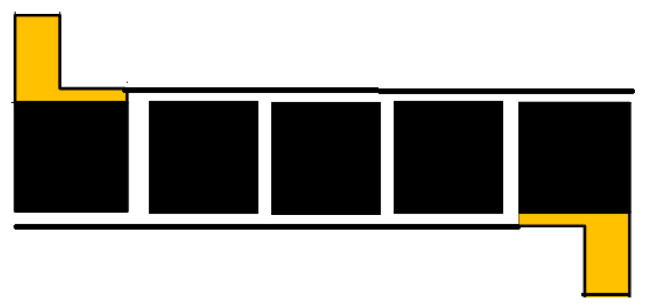
The components of the sensor.

The fabrication of the tactile sensor involved the stacking of three fabricated fabric plates with reconstituted graphene between a laser-induced graphene blank on foil-coated polyimide. Figure 3 shows the components of the sensor in a schematic.

**Figure 4:**
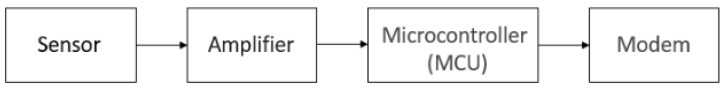
Information processing and transmission diagram.

The electronic unit of information processing and transmission consists of an amplifier, microcontroller, and ZigBee modem, which performs data transfer. Figure 4 shows the schematic diagram of the electronic block of information processing and transmission.

**Figure 5:**
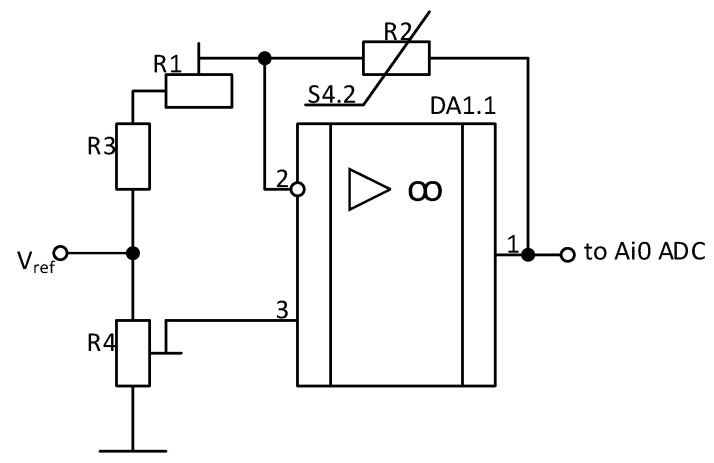
Amplifier circuit diagram.

Figure 5 shows the circuit diagram of the amplifier.

As demonstrated in the Figure, the output voltage of the bridge amplifier circuit is proportional to the relative increment in resistance of the variable resistor. At the output of the amplifier, zero voltage will be observed when the voltages at both inputs of the amplifier are equal to each other. This occurs when the resistance ratio of the resistors is (1).

(1) $$\frac{R_{2}}{R_{1}+R_{3}}=\frac{R_{4.1}}{R_{4.2}}$$

where *R*_4.1_ is the upper part of the adjustable resistor *R*_4_ relative to the moving contact, and *R*_4.2_ is the lower part of the adjustable resistor *R*_4_ relative to the moving contact. Resistor *R*_2_ is a tactile sensor.

Applying pressure to the subject results in a decrease in resistance, a disturbance to the bridge equilibrium, and an amplifier output voltage that is proportional to the applied load. The magnitude of the amplifier output voltage can be controlled by changing the value of the reference voltage Vref.

**Figure 6:**
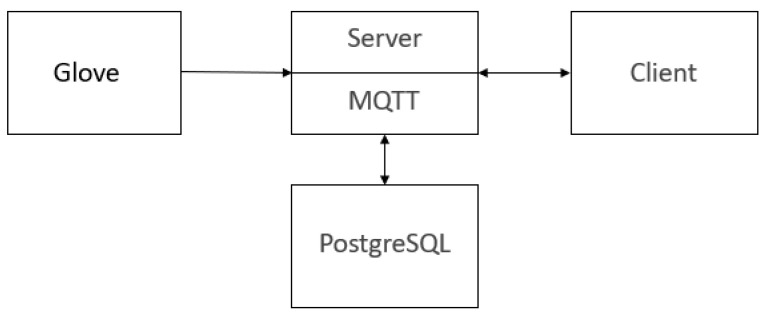
Block diagram of the software service to operate the glove.

The program for the microcontroller (MC) MSP430G2553IPW is completed in Assembler language. This computer programme was designed to function with the device, utilising a battery power supply. Furthermore, approximately half of the time, the MCU operates in low-power-consumption mode (i.e., sleep mode). The MCU operation algorithm is based on interrupt processing from timers TA0 and TA1. Timer TA1 sets the frequency of voltage measurement at the ADC inputs, and timer TA0 determines the rate of transmission of information on the UART interface from the controller to the modem. Thus, every 10 msec, i.e., 100 times per second, there is a polling of 8 ADC channels and received data are transferred to the ZigBee modem for further transmission and processing. Figure 6 shows a block diagram of the software service to operate the glove.

The device is able to communicate via the server, which functions as the intermediary between the device and the external system. The server receives data from the ZigBee modem, processes them, and then transmits them to a client capable of interpreting the received values via the MQTT protocol. The received data are stored in a PostgreSQL database.

A maintenance and repair glove is defined as a glove composed of any material. The distinguishing feature of this glove lies in the integration of tactile sensors beneath each finger pad. These sensors are designed to measure the force exerted during pressure application. The glove is constructed from a composite material combining laser-induced and reconstructed graphene, which facilitates rapid recovery after pressure application. This feature ensures the glove’s readiness to receive signals of any frequency. The sensors installed on the glove are connected by a pair of wires to an electronic unit for the processing and transmission of information. The electronic processing unit and wireless network (ZigBee)’s transmission of information is related to the results interpretation unit, which is capable of correlating the data coming from sensors with a certain stage and a certain type of repair and maintenance work. ZigBee was chosen as a wireless data channel for specific purposes. Firstly, it provides the necessary data transmission speed, as indicated in studies [46,47]. Secondly, using ZigBee, it is possible to accurately estimate the distance from the signal receiver to the object [48,49]. Subsequently, that information will be utilised as an additional parameter to facilitate more precise identification of the actions of service personnel during service.

## 3. Results and Discussion

**Figure 7:**
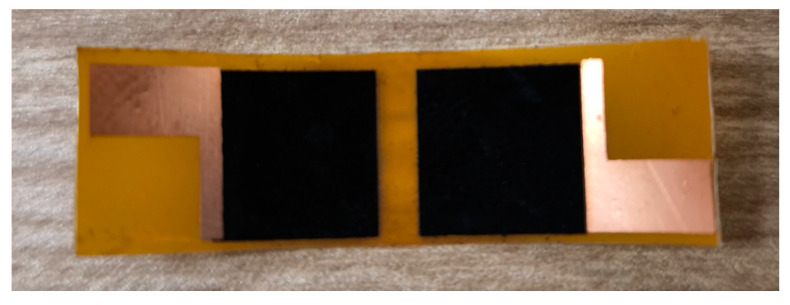
The first sensor part (LIG).

Figure 7 shows the overview of the first sensor part (LIG) placed in a glove.

Copper wires were soldered onto the foil part of the polyimide. To estimate the sensor performance, the resistance between graphene and the foil part of polyimide was measured. During the initial phase of the sensor’s fabrication, two issues were identified in the laser engraving process. Firstly, a burn spot was observed on the polyimide foil during the engraving process. Secondly, there was an absence of contact between the graphene and the polyimide foil component.

**Figure 8:**
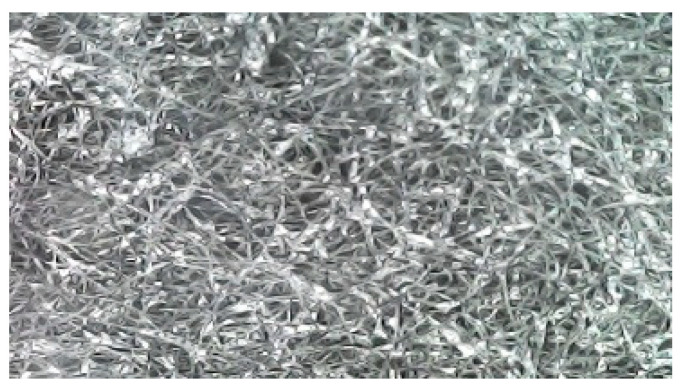
The second part of the tactile sensor (rGO), zoomed in through a digital microscope (1600×).

**Figure 9:**
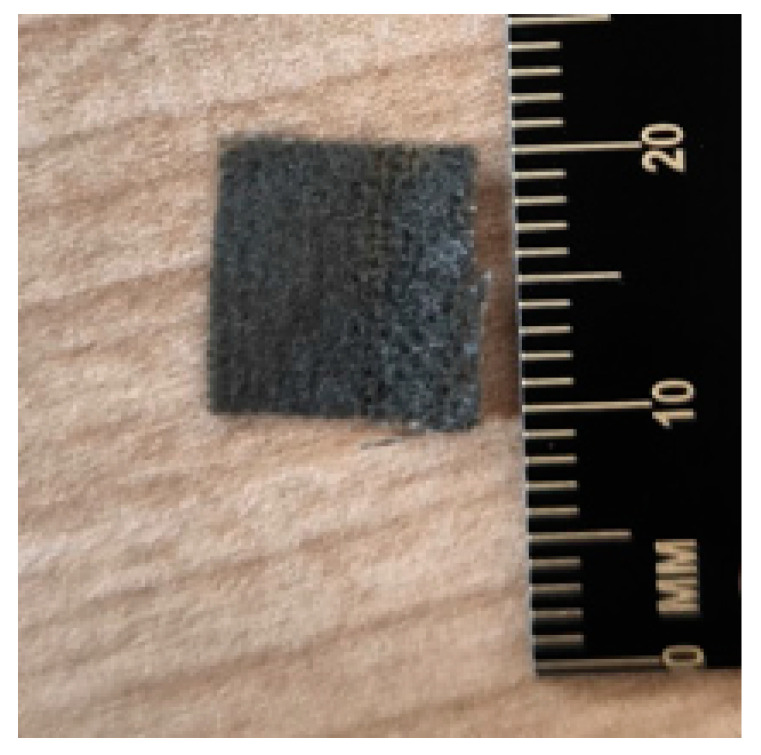
The second part of the tactile sensor (rGO), located in close proximity to a ruler.

Figure 8 shows the second part of the tactile sensor (rGO). There are fabric squares with reconstituted graphene after calcining in the furnace. In order to determine the correct operation of the sensor, the following actions were performed: firstly, the uniform coverage of the pile fabric with graphene was evaluated through the use of a microscope, and secondly, the resistance on each piece of fabric used in the fabrication of sensors was checked with a multimeter. The measurements were made in random places on the entire surface, and their stability indicated the suitability of the obtained material as the second part of the tactile sensor (rGO). The obtained fabric is illustrated in Figure 8 and Figure 9.

The microscope zoom-in on the structure of the fabric demonstrates a uniform distribution of graphene. Thus, it is suitable for sensor performance. The primary challenges encountered during the production process are as follows: inadequate soaking in graphene oxide, calcination, and the attainment of an uneven distribution of graphene on the textile.

**Figure 10:**
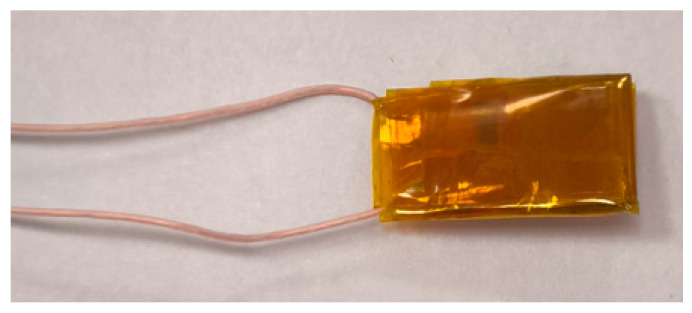
The tactile sensor.

Figure 10 shows the overview of the obtained tactile sensor.

The primary concern regarding the sensors obtained is the instability of the resistance. It is imperative to conduct a series of additional studies to obtain stable characteristics. To address this challenge, a customised bridge circuit with an integrated adjustable resistor has been employed in the present study. Table 1 shows the characteristics of the obtained sensors and the peculiarities of their connection to the microcontroller.

**Figure 11:**
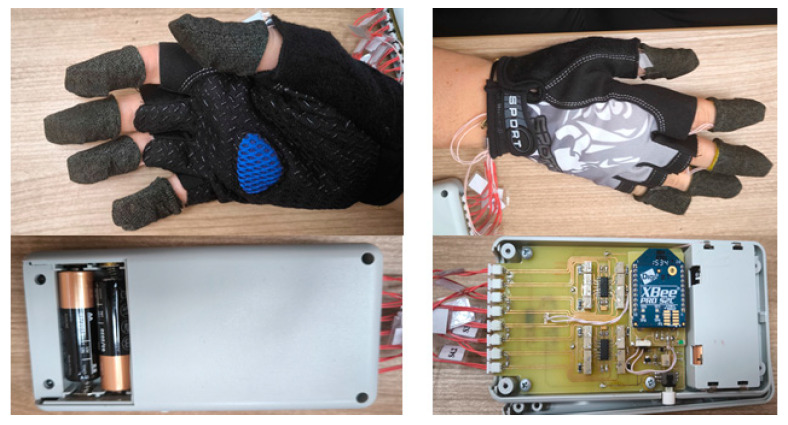
Smart glove.

Figure 11 shows an image of the resulting device.

The electronic unit can be attached on the arm, in a special pocket. The weight of the electronic unit together with batteries is 165 g.

**Figure 12:**
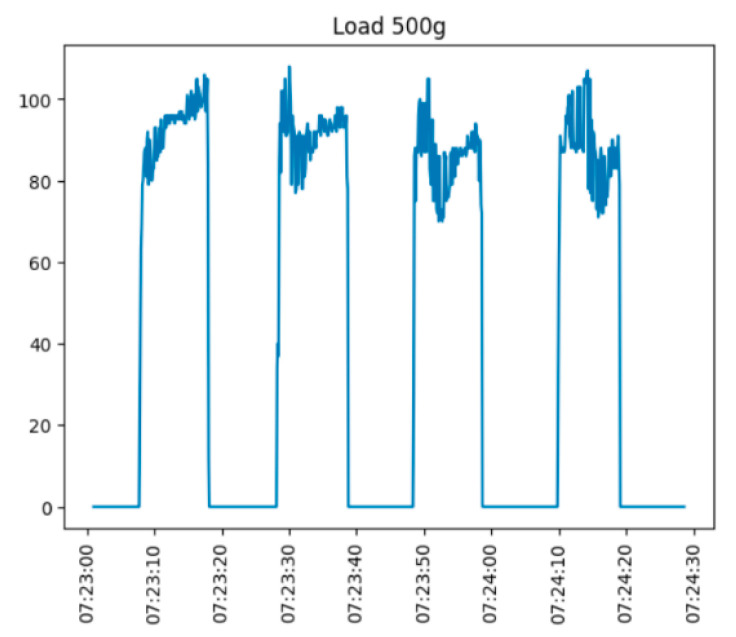
Time variation of the sensor output signal (load—500 g).

**Figure 13:**
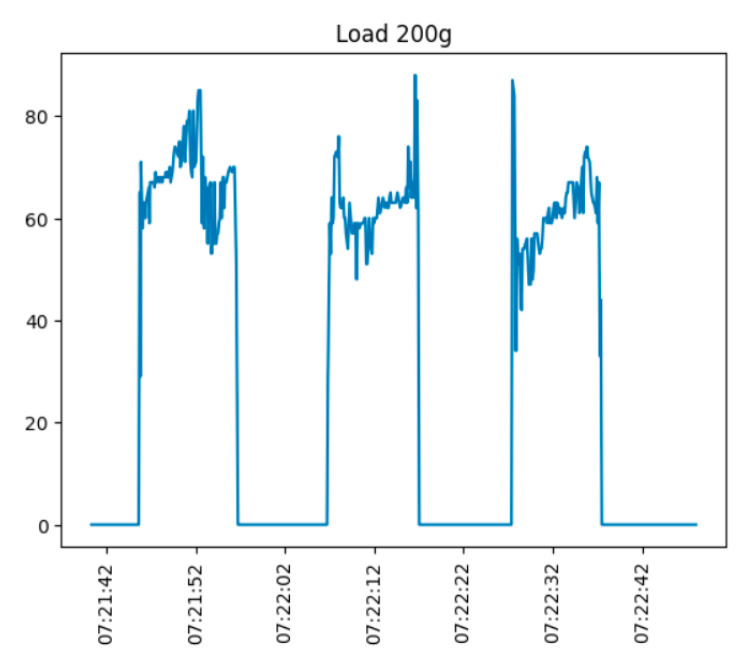
Time variation of the sensor output signal (load—200 g).

Figure 12 and Figure 13 demonstrate the sensor testing under load. Weights of 200 and 500 g were placed on the sensor at any moment and removed. The experiment was repeated 3–4 times.

As can be seen from the figure, the sensor operates fine under the applied load and quickly returns to the initial state. However, the sensor signal is very noisy. In addition, the stable characteristic of the maximum voltage of the output signal is not visible. This is primarily associated with the weight being set in manual mode, resulting in oscillations, which are particularly strong during the setting and removal of the weight.

**Figure 14:**
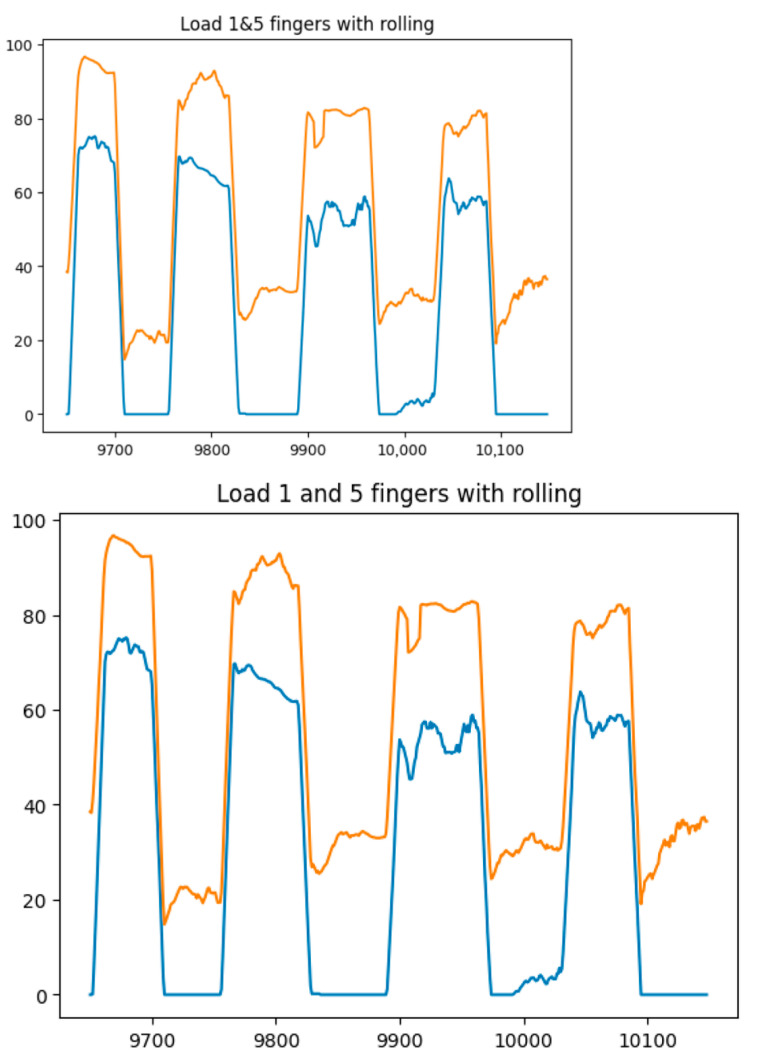
Graphs of alternating finger closings (1 (blue line) and 5 (orange line) fingers).

**Figure 15:**
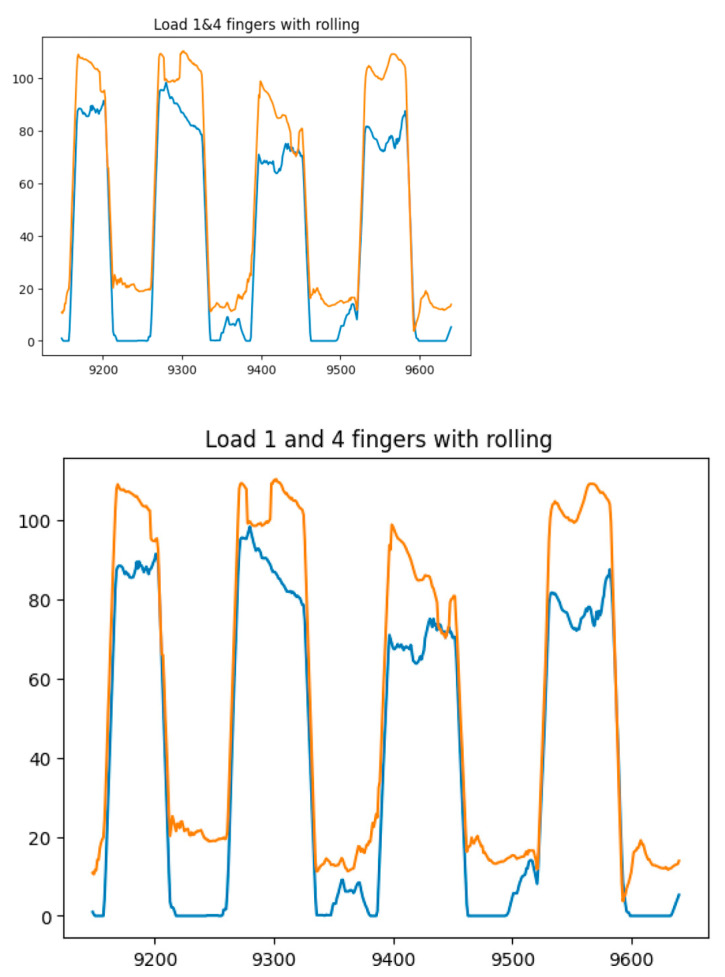
Graphs of alternating finger closings (1 (blue line) and 4 (orange line) fingers).

**Figure 16:**
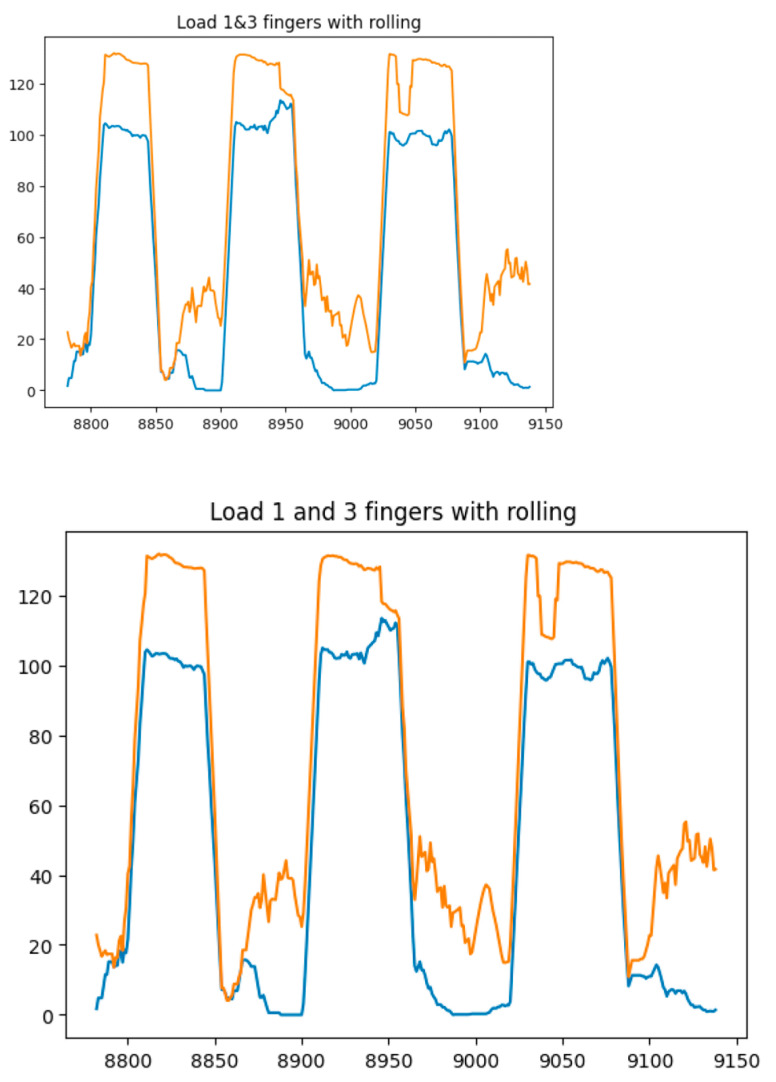
Graphs of alternating finger closings (1 (blue line) and 3 (orange line) fingers).

**Figure 17:**
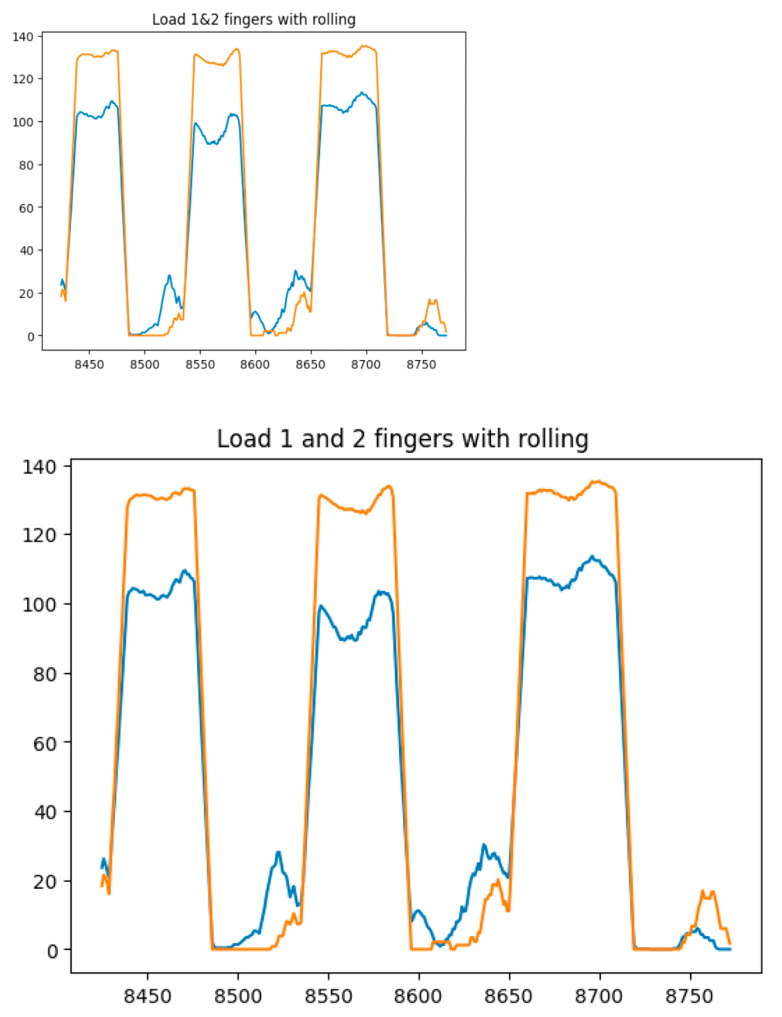
Graphs of alternating finger closings (1 (blue line) and 2 (orange line) fingers).

Figure 14, Figure 15, Figure 16 and Figure 17 show the graphs of the experiment of alternate finger closing. The obtained output signals were filtered by the moving average method with a period window of 10.

**Figure 18:**
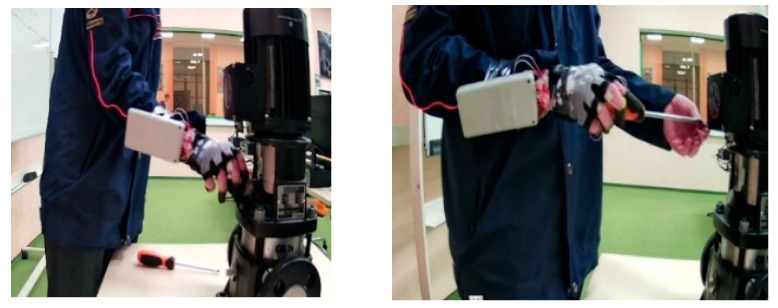
Maintenance and repair with the smart glove.

Figure 18 shows the changes when the fingers are in closed and relaxed positions. In this way, the sensors react properly to the events that occur. The obtained results show that the developed device can be applied for technical repair and maintenance of personnel.

Moreover, the device was additionally utilised for the maintenance of the centrifugal pump, a process which entailed the removal of the protective casing and the disassembly of the pump. The unit was designed to capture information during the repair process and to evaluate the information capacity of the system.

The maintenance and repair video was recorded with a Xiaomi Xiaovv HD web camera and processed on a Rockchip RK3588 microcontroller [50]. In total, we obtained three videos of 5 min duration, and their sizes were 37.2 MB, 28.3 MB, and 25.5 MB. The size of the data file was 195 kB. The data storage requirements for the equipment’s repair operations were significantly smaller than the video data. This is undoubtedly a benefit of the proposed technology.

There are several points that need to be discussed further.

1. Comparison of the proposed smart glove with the existing wearable sensors used for maintenance. A comparison of different wearable devices used in the performance of maintenance and repair can include glasses, smart clothes, insoles and boots, bracelets, smart helmets, and wearable video cameras. Smart gloves can be used as both an independent device to manage maintenance and repairs, and as part of a comprehensive system of wearable devices. With regard to the listed devices, the functions of eyeglasses and wearable video cameras are the most analogous. The authors have developed a system that allows the determination of the stages of work in the maintenance process from the video image. The analysis of the video stream enables the estimation of the action performance by the operator. However, the use of gloves for this purpose provides additional information. The degree of pressure applied can be deduced, and the effort expended during the maintenance operation can be assessed. These parameters can be indirectly compared with the action operator quality of the maintenance. It is challenging to achieve this through video stream processing alone. However, it is important to emphasise that the integration of multiple wearable devices into a unified system will undoubtedly generate more substantial results. But further research is needed to prove this.
2. The impact of signal noise and instability in the sensor data. Noise and instability of the sensor’s signals has the potential to distort the information. The simplest methods for noise reduction, such as the moving average, have been employed in this study. As demonstrated by the experimental findings, this approach is adequate for achieving stable characteristics. Nevertheless, further research is required in this area to explore the full potential. It could be that using machine learning, wavelet transform, and other methodologies for noise reduction would have given better results. The application of machine learning, wavelet transform, and other methodologies holds promise in the development of enhanced noise reduction systems. Furthermore, machine learning models have the ability to filter noise themselves. Additionally, using this model, we should not denoise the signal. Also, machine learning models will be capable of addressing short-term information gaps from sensors. These issues require further investigation.
3. Testing of the sensors at high temperatures or in humid conditions. It is imperative to assess the sensor’s performance under conditions of humidity and elevated temperatures prior to its induction into production. However, this is not currently feasible. Even in the absence of testing, it can be stated that the sensor without the protective layer will be incapable of operating in humid conditions. The sensor’s design parameters preclude such functionality. However, since the sensor is placed inside a special material that forms a glove, this material allows the sensor to remain functional. A series of experiments were conducted with gloves, putting them on for several hours, which demonstrated the efficacy of the system. However, it is crucial to acknowledge the necessity of future research in this area, as the findings can significantly impact the feasibility of integrating this sensor within the glove. The authors of the study emphasise the importance of this consideration. However, according to the authors’ point of view, all these problems can be solved by using a special material for gloves.
4. The challenges of scaling production of this smart glove. The presented technology has the capacity to be scaled up for any number of production facilities and any number of service personnel. When scaling this technology individually, gloves are manufactured for each person, as devices 1, 2, and 4 according to Figure 1. The results interpretation unit can be used for all devices, provided that the ZigBee network is properly organised. The necessity for the installation of the results interpretation unit for each individual device is negated. From an architectural standpoint, the results interpretation unit can be redundant and can be integrated with third-party systems.
5. Ensuring data privacy. The security of the device should be examined independently. It is important to note, however, that in the context of wireless communication, achieving absolute security is impractical. The principles of ensuring information security of the device are analogous to the ZigBee network. Despite the inclusion of separate devices, testing for cybersecurity in subsequent studies is recommended.

## 4. Conclusions

The results obtained generally demonstrate the applicability of the smart glove for equipment maintenance and repair. Graphene-based sensors have been shown to rapidly detect pressure and swiftly revert to their original state. The durability of graphene, a critical property, has been demonstrated as effective in the context of the tests conducted. Nevertheless, further experimentation is required to ascertain the reliability of the technology outlined in this paper and to determine the accuracy of the measurements obtained. It is evident that the circuit has been designed with a specific set of characteristics in mind, and that any variation would necessitate recalibration. In the event of a deviation from the original characteristics, the circuit provides a possibility of calibration with the help of adjustable resistors. However, it is still necessary to calibrate the scheme. In order to identify the actions of repair personnel and the signals received from the glove, it is essential to perform experiments, collect training and test data, and set up machine learning models. Undoubtedly, this necessitates further research. The present study examined the speed and volume of information acquisition, response to events, and reliability. The following research will be related to the development of technology to obtain unified stable output characteristics of all sensors, improve their strength and reliability characteristics, further the development of machine learning algorithms for the identification of personnel actions, and check the quality of data transmission channel performance. Following the successful attainment of stable results for each part of the sensor, the authors discovered that when the sensors were connected into a single structure, the resistance was different and stability could not be achieved. This indicated that the method of fixing the three parts of the sensor was the problem. Subsequent research will be directed towards the identification of a bonding material and the development of a design that facilitates more precise fixation of the sensor components. The focus of this study is on hydrogels capable of self-healing [51] and materials that create an ultra-transparent conductive layer [52]. Of particular interest are materials that both modify their electrical parameters and possess the capacity to generate electrical energy (materials with a triboelectric effect [53]). Irrespective of the sensor employed, the fundamental principles of the proposed device will be maintained, provided that the sensor quantifies the degree of pressure and, either autonomously or via the primary processing unit, transmits the collected data to the block responsible for interpreting the results. A machine learning model will be created for the results interpretation unit. It needs many series of experiments. Two distinct model types will be developed: a model that is trained on examples of repair actions (referred to as Supervised Learning) and a model that enables data clustering independently (Unsupervised Learning). In both cases, datasets will be trained. The creation of these datasets will be informed by the execution of the simplest repair actions for a centrifugal pump. The amount of data collected will ensure effective learning. Subsequent to the accumulation and marking up of the data, the model will be subjected to training and tested in real-world conditions. To simplify data marking up, a system will be used to separate repair and maintenance steps by video sequence. Additionally, an unsupervised model will be trained on these data. The testing of the resulting models will be conducted in parallel, with the same actions being performed with the pump under laboratory conditions. If successful, the pump maintenance actions will be rendered more complex, and the number of objects and tasks will be increased. The computing power utilised to operate the system and the size of the data employed will be assessed, as well as the system’s capacity to generate reports on the operations performed and the repair of quality losses in the execution of work. These may include incorrect sequences of operations, insufficient force, e.g., tightening bolts, and so forth. Furthermore, the investigation will encompass the cognitive–psychological state of personnel during glove utilization, its suitability and effectiveness, and the consideration of glove efficacy when employed in conjunction with augmented and virtual reality systems.

## Figures and Tables

**Table 1:** Connecting tactile sensors to a microcontroller.

Sensor	Sensor Resistance, MOm	Resistance of Additional Resistor, MOm	Out of MCU	ADC Channel	ID
S1.1	7.5	6.2	14	AI6	0Bh
S1.2	10.2	10	15	AI7	0Ch
S2.1	6.7	6.2	7	AI5	15h
S2.2	6.3	6.2	6	AI4	16h
S3.1	9.2	9.1	5	AI3	1Fh
S3.2	10.4	10	4	AI2	20h
S4.1	2.2	2	3	AI1	29h
S4.2	5	4.7	2	AI0	2Ah

## Data Availability

Data are contained within the article.
